# Revisiting the Hydrogen Storage Behavior of the Na-O-H System

**DOI:** 10.3390/ma8052191

**Published:** 2015-04-28

**Authors:** Jianfeng Mao, Qinfen Gu, Duncan H. Gregory

**Affiliations:** 1 WestCHEM, School of Chemistry, Joseph Black Building, University of Glasgow, Glasgow G12 8QQ, UK; E-Mail: jeff.mao@hotmail.com; 2 Australian Synchrotron, Clayton, Victoria 3168, Australia; E-Mail: Qinfen.Gu@synchrotron.org.au

**Keywords:** hydrogen storage, sodium oxide, sodium hydride, sodium hydroxide, *in-situ* synchrotron powder diffraction

## Abstract

Solid-state reactions between sodium hydride and sodium hydroxide are unusual among hydride-hydroxide systems since hydrogen can be stored reversibly. In order to understand the relationship between hydrogen uptake/release properties and phase/structure evolution, the dehydrogenation and hydrogenation behavior of the Na-O-H system has been investigated in detail both *ex-* and *in-situ*. Simultaneous thermogravimetric-differential thermal analysis coupled to mass spectrometry (TG-DTA-MS) experiments of NaH-NaOH composites reveal two principal features: Firstly, an H_2_ desorption event occurring between 240 and 380 °C and secondly an additional endothermic process at around 170 °C with no associated weight change. *In-situ* high-resolution synchrotron powder X-ray diffraction showed that NaOH appears to form a solid solution with NaH yielding a new cubic complex hydride phase below 200 °C. The Na-H-OH phase persists up to the maximum temperature of the *in-situ* diffraction experiment shortly before dehydrogenation occurs. The present work suggests that not only is the *inter*-phase synergic interaction of protic hydrogen (in NaOH) and hydridic hydrogen (in NaH) important in the dehydrogenation mechanism, but that also an *intra*-phase H^δ+^… H^δ–^ interaction may be a crucial step in the desorption process.

## 1. Introduction

With the continued depletion of fossil fuel resources and the increasing impact of environmental pollution, renewable and clean energy sources such as wind and solar technologies have become a major priority and been the subject of increased research interest. However, many renewable energy sources are intermittent and so a means by which energy can be stored and transported is vital [1]. One approach is to store energy chemically as a clean fuel and hydrogen is regarded as one of the best options due to its abundance, high gravimetric energy density and capacity to be sustainably generated. However, a means to store hydrogen safely at high capacity and low cost is key to its successful implementation [2,3].

Compared to storage of hydrogen as a compressed gas or a cryogenic liquid, solid-state hydrogen storage is more effective volumetrically and alleviates safety and cost concerns associated with high pressure and/or low temperature. For more than 10 years materials such as light metal hydrides [4], complex hydrides [5,6,7] and chemical hydrides [8] have been investigated in this capacity and significant advances in understanding and performance have been made. However, no single hydride system yet fulfills all the necessary criteria for mobile applications (principally gravimetric and volumetric capacity, sorption enthalpy and kinetics) [9]. When compared to mobile applications, the requirements for stationary applications are rather different and cycle life/longevity, cost and safety can become the most important parameters [10]. Given the technical demands for portable storage, static medium-large scale hydrogen storage may be at the forefront of initial efforts in energy storage and in shifting electrical energy from peak to off-peak periods to achieve smart grid management. For this purpose, an abundant, low cost and non-toxic hydrogen storage material becomes increasingly attractive to ensure large-scale and long-term applications with the minimum of financial burden.

Among various hydrogen storage materials, sodium based materials are very promising as hydrogen storage media for stationary applications since sodium is one of the most abundant elements on Earth (2.64 wt%), and is relatively cheap [11,12,13,14,15]. Moreover, sodium resources are geographically ubiquitous (e.g. from the sea and from underground deposits). Recently, Xu *et al*. found that sodium oxide, Na_2_O can absorb hydrogen easily at close to ambient temperature (~60 °C) to form NaH and NaOH. Further, the hydrogenated products, NaH and NaOH, can be readily converted back to Na_2_O by thermal treatment [16,17]:

Na_2_O + H_2_↔NaH + NaOH  ∆H = 55.65 kJ/mol H_2_ (3.2 wt%) (1)

Dehydrogenation in the NaH-NaOH system occurs at a lower temperature than that for NaH alone and given that NaOH and NaH contain H^δ+^ and H^δ–^, respectively, the interaction of these two hydrogen species could be responsible for the relative decrease in dehydrogenation temperature between Na-O-H and Na-H [16,17]. The dehydrogenation process and mechanism are still not clear, however. Interestingly a number of much earlier studies of the Na-O-H system have indicated that the alkali metal hydride and corresponding hydroxide may be miscible [18,19,20,21,22]. More recently, during a study of the effect of NaOH as an additive in the Na-H system, it was observed that *ca*. 10 mol% of NaOH could apparently be incorporated into the NaH structure and resulted in enhanced hydrogen motion in NaH above 150 °C [23]. These previous results suggest that OH^–^ can be substituted for H^–^ within the NaH structure above 150 °C. Given the connectivity between proton conduction and hydrogen uptake/release kinetics [24], an investigation of the structural and phase composition changes in the Na-O-H system during heating should provide considerable insight into the desorption mechanism and ultimately in how the performance of the system might be improved. In this paper, we present a detailed study of hydrogenation and dehydrogenation in the Na-O-H system, particularly exploiting *in-situ* synchrotron X-ray powder diffraction methods to elucidate the subtle changes in composition and structure immediately prior to hydrogen release.

## 2. Results and Discussion

### 2.1. Hydrogenation of Na_2_O

**Figure 1:**
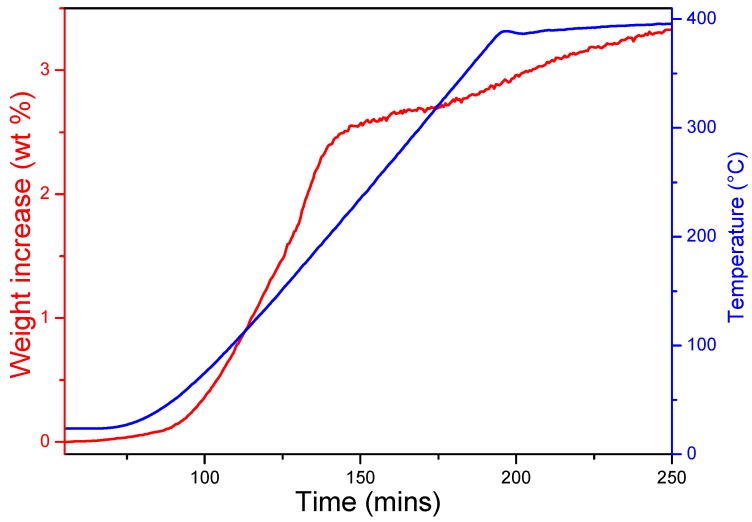
Hydrogen uptake of as-milled Na_2_O under 18 bar of hydrogen.

The time-resolved hydrogenation profile of as-milled Na_2_O with temperature (at 18 bar) is shown in Figure 1. On heating at 3 °C min^−1^, hydrogen uptake is initiated close to room temperature (30–50 °C). Upon heating to 140 °C, the hydrogen uptake rate increases significantly and 2.5 wt% H_2_ can be stored under these conditions. Subsequently, hydrogenation is relatively slow and a total of 3.3 wt% hydrogen is absorbed by 400 °C. These uptake results are consistent with previous studies [16,17], although it should be noted that the experimental figure of 3.3 wt% slightly exceeds the theoretical capacity. In principle, this could be possible due to the partial reaction of Na_2_O with moisture during hydrogen uptake, but powder X-ray diffraction (PXD) patterns before and after hydrogenation suggest that the reaction of an impurity in the starting material with hydrogen may also contribute as discussed below.

**Figure 2:**
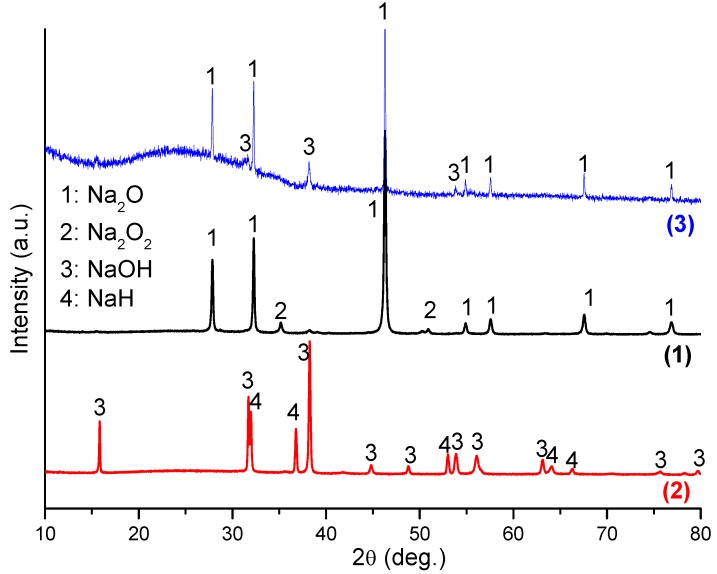
XRD patterns of Na_2_O after ball milling (sample 1), after hydrogenation at 400 °C/18 bar H_2_ (sample 2), and after subsequent dehydrogenation at 400 °C (sample 3).

PXD was performed to clarify the chemical reactions that occur on hydrogenation. Figure 2 shows the PXD patterns of the Na_2_O starting material after mechanical milling (sample 1), the material after hydrogenation (sample 2) and finally after subsequent dehydrogenation (sample 3). Although Na_2_O was the main phase in the ball milled starting material as expected, an impurity phase of Na_2_O_2_ was also detected, and analysis of the diffraction pattern yielded a phase fraction of *ca*. 10 wt%. After hydrogenation at 400 °C, NaH and NaOH were formed and the diffraction peaks from Na_2_O and Na_2_O_2_ were no longer present. Upon dehydrogenation at 400 °C, Na_2_O reformed and was present as the majority phase. Overall, therefore, the material system demonstrated a reversible reaction as described by Equation 1. The presence of NaOH in the dehydrogenated product, sample 3 could suggest partial hydrolysis of Na_2_O, but the more likely origin of the hydroxide is from hydrogenation of the original Na_2_O_2_ impurity in the starting material (since Na_2_O_2_ + H_2_→2NaOH). This leads to an excess of NaOH in the hydrogenated product, sample 2 (*i.e*., NaH:NaOH is not in the expected 1:1 molar ratio following hydrogen uptake) which persists during the dehydrogenation process at 400 °C However, Rietveld refinement for hydrogenated Na_2_O shows that the molar ratio of NaOH and NaH in the sample is 1.66:1. The phase fraction of NaOH exceeds the amount expected solely from the hydrogenation of 10 wt% Na_2_O_2_. Thus, both the hydrolysis of Na_2_O (and possibly of NaH) and the hydrogenation of the original Na_2_O_2_ impurity lead to the observed excess of NaOH.

### 2.2. Dehydrogenation of NaH-NaOH

**Figure 3:**
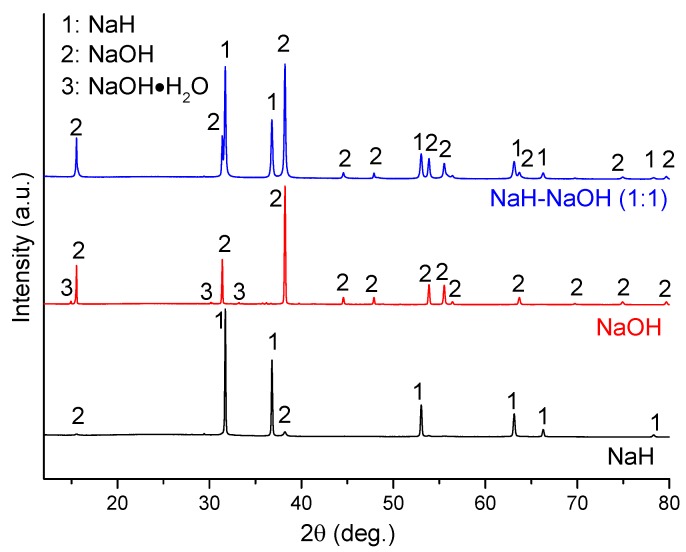
PXD patterns of as-received NaH and NaOH, and as-milled NaH-NaOH (1:1), sample 4.

The dehydrogenation process in the NaH-NaOH system was investigated further. Figure 3 shows a PXD pattern of the as-milled mixture of NaH-NaOH (molar ratio 1:1; sample 4) as compared with the individual commercial starting materials NaH and α-NaOH. It can be seen that the as-received NaH contains a small amount of NaOH impurity, while the as-received NaOH contains a minor phase of the hydrated hydroxide, NaOH·H_2_O. By comparison, only NaH and NaOH were detected in sample 4. The absence of the NaOH·H_2_O impurity in sample 4 is attributed to dehydration during ball milling.

**Figure 4:**
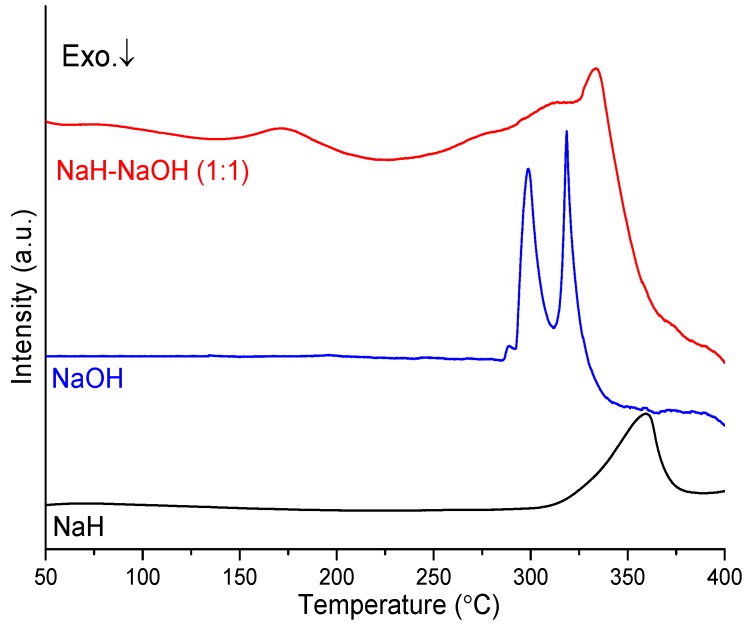
DTA profiles of NaH, NaOH, and as-milled NaH-NaOH (1:1) mixture.

The thermal decomposition behavior of sample 4 compared to NaH and NaOH was investigated by DTA, as shown in Figure 4. The DTA profile for NaH shows one endothermic peak at *ca*. 360 °C, which can be assigned to the decomposition of NaH to Na metal and hydrogen. For NaOH, the DTA profile shows two endothermic peaks at 299 and 319 °C, which can be assigned to the α-β (orthorhombic-monoclinic) phase transition and the melting point, respectively [25,26]. By contrast, sample 4 shows different features, displaying multiple endothermic peaks. The first endothermic event occurs at 171 °C and is relatively well-defined while the second is more complex (consisting of perhaps three or more individual processes) and reaches a maximum in the DTA profile at 333 °C. The second endothermic event can be attributed to the reaction of NaH and NaOH culminating in the formation of Na_2_O and hydrogen. The results further confirmed that the dehydrogenation pathway of the NaH-NaOH mixture is entirely different to that observed for NaH. The first endothermic event is not observed in either NaH or NaOH. It would appear therefore that the synergic interaction of hydrogen species in NaH and NaOH not only contributes to the lower dehydrogenation temperature *vs*. NaH itself, but also leads to likely reaction in the solid state and structural changes during the heating period prior to dehydrogenation. To clarify the structural changes that occur before dehydrogenation, variable temperature, *in-situ* synchrotron PXD experiments were conducted on the NaH-NaOH mixture and are discussed below.

**Figure 5:**
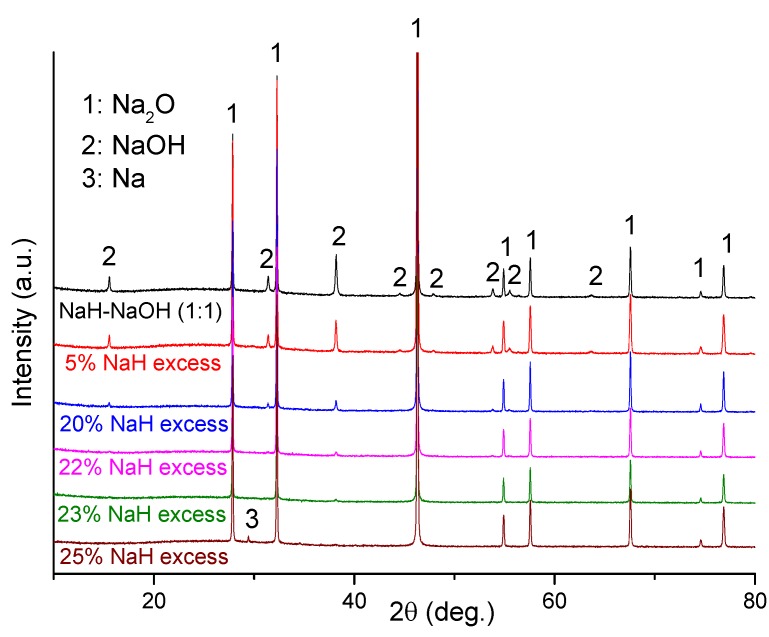
PXD patterns of NaH-NaOH with varying hydride:hydroxide molar ratio after dehydrogenation at 400 °C.

*Ex-situ* XRD characterization was performed on several dehydrogenated hydride-hydroxide mixtures of varying molar ratio after heating each to 400 °C (Figure 5). Clearly, Na_2_O was the main phase in the dehydrogenated NaH-NaOH (1:1) sample, but some residual NaOH remains also detected. In this case, the presence of residual NaOH can be attributed to the fact that the NaH and NaOH starting materials contain low levels of NaOH and NaOH·H_2_O impurities, respectively. Hence the NaH:NaOH ratio departs from the ideal stoichiometric 1:1 value in the mixture. In order to obtain single phase Na_2_O as a dehydrogenation product, we optimized the quantity of NaH and NaOH in the starting mixture. As shown in Figure 5, NaOH impurity reflections diminished as the NaH:NaOH ratio increased, reaching a minimum when a 23 wt% excess of NaH was used. On adding a 25 wt% excess of NaH, no NaOH was observed in diffraction patterns but reflections originating from Na metal appeared. Therefore, use of 23 wt% excess of NaH was found to be the optimum reactant composition required to obtain Na_2_O with minimal impurities (sample 5).

**Figure 6:**
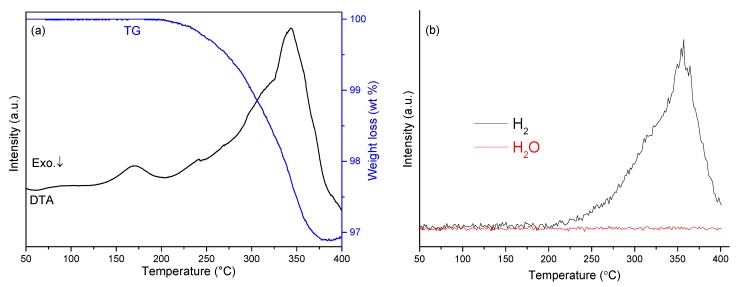
Simultaneous TG-DTA-MS profiles of sample 5: (**a**) TG-DTA traces; (**b**) mass spectra for hydrogen and water.

The thermal behavior of sample 5 was investigated by TG-DTA-MS (Figure 6). TG results showed that the mixture released gas from 200–378 °C with a corresponding weight loss of 3.1 wt%. Moreover, it was evident from mass spectra collected simultaneously while heating that hydrogen was the only evolved gaseous product, consistent with the reversible reaction described by equation 1. The DTA profile for sample 5 reveals a similar feature with the sample NaH-NaOH (1:1). The first endothermic peak was observed at 170 °C, while the second cluster of overlapping endothermic peaks with a maximum at 342 °C is attributed to the dehydrogenation reaction of NaH with NaOH and might be expected to incorporate first the α-β phase transition and second the melting of NaOH.

### 2.3. In-Situ Synchrotron Powder Diffraction

**Figure 7:**
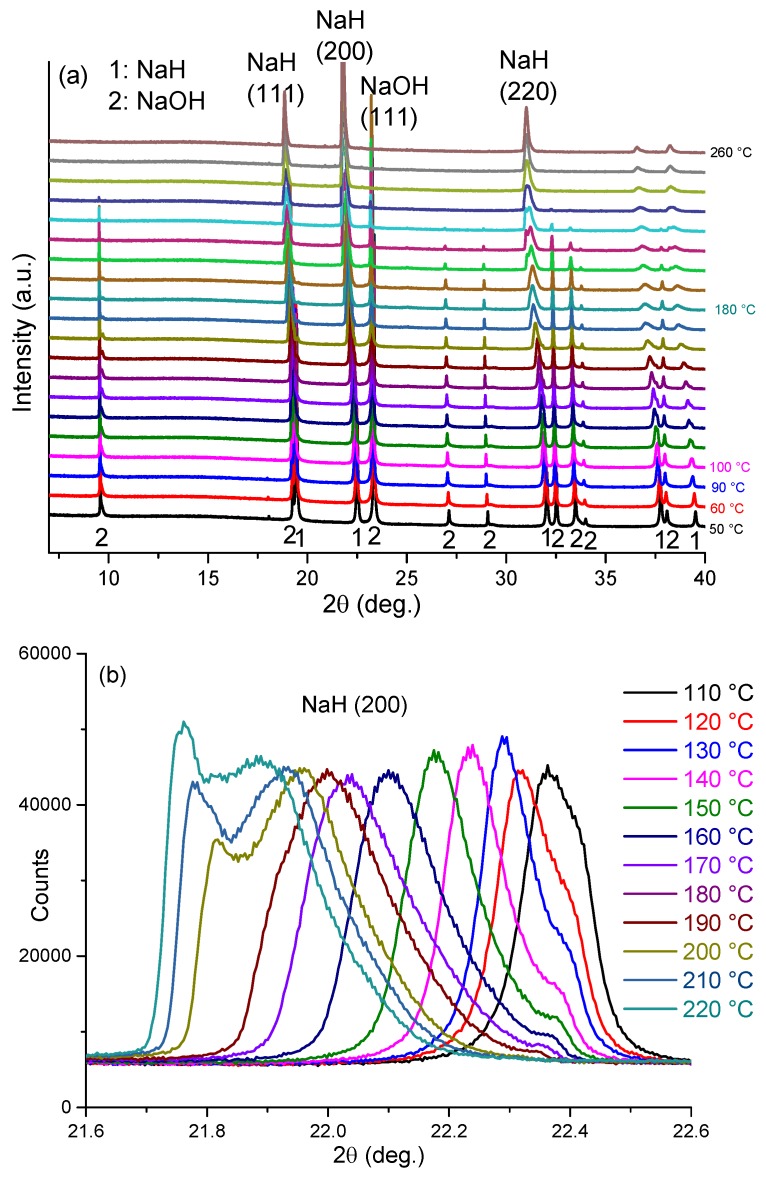

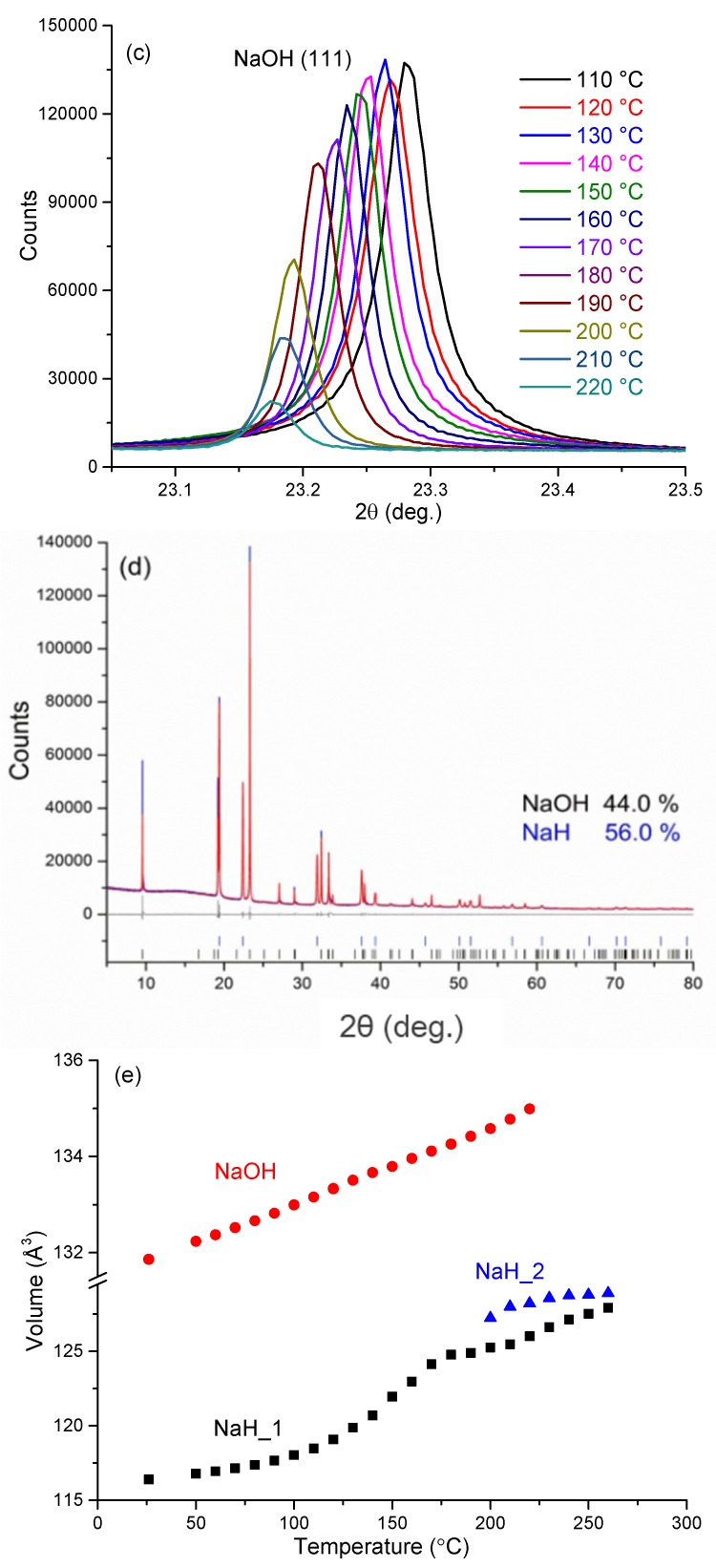
*In-situ* synchrotron PXD patterns for sample 5 heated from room temperature to 260 °C with a constant heating rate of 10 °C min^–1^ in a closed quartz capillary under Ar atmosphere (λ = 0.9533 Å) showing: (**a**) the region from 7° ≤ 2θ≤ 40°; (**b**) the change in the NaH (200) reflections from 110 to 220 °C; (**c**) the change in the NaOH (111) reflections from 110 to 220 °C; (**d**) the refinement profile at 100 °C; and (**e**) cell volume against temperature for NaH (“NaH_1”), “Na-O-H” (“NaH_2”) and NaOH, respectively, from room temperature to 260 °C.

*In-situ* synchrotron PXD methods were used to clarify the reaction mechanism underpinning the first endothermic event at *ca*. 170 °C by heating sample 5 from room temperature to 260 °C. Figure 7 shows selected regions of the *in-situ* diffraction patterns for sample 5, the relevant peak indices and a plot of the resulting cell volume changes of the NaH and NaOH phases, respectively, as a function of temperature. Structure refinements performed against the synchrotron PXD data were conducted for each data set (see supplementary information). A fundamental parameter (FP) approach was employed in TOPAS to perform whole-pattern profile fitting of the diffraction data collected in transmission mode. The diffraction background was fitted with Chebychev functions. Structural data from the ICDD PDF4 (2014) database for NaH (02-0809) and α-NaOH (078-0188) were used as starting models in TOPAS. At room temperature NaH crystallizes in cubic space group *Fm**3**m* (No.225; *a* = 4.8826(1) Å), while α-NaOH is orthorhombic (*Cmcm*, No. 63; *a* = 3.4039(1) Å, *b* = 3.4011(1) Å, *c* = 11.3901(1) Å).

Before heating, room temperature PXD data show that sample 5 consists only of NaH (44 wt%) and NaOH (56 wt%). There are no impurity diffraction peaks observed in the PXD pattern. As shown in Figure 7, below 110 °C, reflections for both NaH and NaOH shift slightly to lower angle with increasing temperature. This shift corresponds to the expected thermal expansion of the NaH and NaOH lattices. The cell volume for NaH increases from *ca*. 116.4 Å^3^ to *ca*. 118.5 Å^3^ over this temperature range, corresponding to a volume expansion of approximately 1.8%. Over the same temperature range, the cell volume for NaOH increases by approximately 0.99% and the phase fractions of NaH and NaOH remain effectively constant, indicating that no reaction occurs between the hydride and hydroxide.

From 110 °C, all the NaH peaks become increasingly asymmetric with some evidence of reflections emerging at slightly higher 2θ values to the main peaks as manifested by a broadening tail to all NaH reflections. Meanwhile, the NaH peaks shift continuously to lower 2θ angles and the hydride cell volume increases significantly such that at 180 °C the value is approximately 7.2% larger compared to that at room temperature (Figure 7e). Most significantly, however, starting from 190 °C, the NaH peaks become asymmetric at lower diffraction angle while simultaneously the NaOH diffraction peaks become weaker. As can be seen clearly for the NaH 200 reflection at 2θ ~ 22.4° (Figure 7b), as the temperature increases a new peak starts to appear at a slightly lower 2θ angle. Given that every NaH peak splits in the same sense and that all the original reflections are also retained, the phenomenon indicates the formation of a slightly larger unit cell with a structure type common to the original NaH phase. This indicates that a new NaH-like phase with slightly larger lattice parameters is formed as NaOH becomes depleted.

By 240 °C there is no evidence of NaOH in the diffraction patterns and the NaH-like phase reflections become more symmetric and sharper, indicative of a single phase. Also noteworthy is that there is no evidence for the formation of Na_2_O over the entire temperature range of the experiment. The cell volume of the hydride at 240 °C is 127.1 Å^3^, which is approximately 9.2% larger as compared to the room temperature NaH structure. The results suggest that a structural change begins from 110 °C and that by 190 °C NaOH reacts appreciably with NaH in the solid state to form a complex hydride with an NaH-type structure. As the temperature increases further, a single composition of the NaH-like phase is formed. Thus we propose that an NaH-NaOH solid solution forms in which up to 50% of the hydride is replaced by hydroxide; NaH_1-x_(OH)_x_ where x ≤ 0.5. Hence, one might reasonably speculate that the formation of the hydride-hydroxide is the vital precursor to an *intraphase* H^δ^… H^δ–^ interaction and the hydrogen evolution step during desorption.

The process could thus be represented by the modified version of the reaction equation below:

NaH + NaOH→2NaH_0.5_(OH)_0.5_→Na_2_O + H_2_ (2)

When compared with other *A*H-*A*OH(*A*OH)_2_ systems (*A* = Li, K, Mg), similar solid solution behavior has only been observed previously in the K-O-H system [19]. On heating, however, KOH-KH does not apparently combine to form K_2_O, but rather KH decomposes independently while KOH remains to 500 °C [17]. By contrast, in the LiOH-LiH system, although the final decomposition reaction product is Li_2_O, no solid solution phases are reported prior to oxide formation upon heating, which might be a consequence of the relatively low dehydrogenation temperature [17,27,28]. There is no strong evidence for solid solution formation in the MgH-Mg(OH)_2_ system prior to dehydrogenation [29]. Na-O-H is unique among these four hydroxide-based combinations as the only system with the appropriate thermodynamics for reversible hydrogen storage. The present work suggests that for hydroxide-hydride materials not only is the inter-phase synergic interaction of protic hydrogen (in NaOH) and hydridic hydrogen (in NaH) important in the dehydrogenation mechanism, but that also an intra-phase H^δ+^… H^δ–^ interaction may be a crucial step in the desorption process. Furthermore, the ensuing lattice expansion and anion disorder of the sodium hydride hydroxide could play a significant role in the diffusion of hydrogen in the solid state either or both as protons and hydride. The mobility of one or both of these species is likely to be key to mediating and controlling the hydrogen uptake and release kinetics in this and similar systems. Strategies involving nanostructuring, additives and catalysts are likely to be crucial in the development of cheap, abundant materials systems such as hydroxides into potentially useful hydrogen stores.

## 3. Experimental Section

Na_2_O (Alfa Aesar, anhydrous, 90%), NaH (Sigma Aldrich, dry, 95%), and (α-)NaOH (Sigma Aldrich, reagent grade, 97%) were used as received. Before the hydrogenation or dehydrogenation experiments, Na_2_O and NaH-NaOH mixtures (employing different NaH:NaOH molar ratios as indicated elsewhere in the text), respectively, were milled using a 50 mL stainless steel milling jar under argon atmosphere in a Retsch PM100 planetary ball mill. Milling was performed at 400 rpm for 1 min in one direction, paused for 1 min and then reversed to give a total milling duration of 1 h. A ball:powder mass ratio of approximately 50:1 was employed throughout. All manipulations were performed in an Ar-filled recirculating glovebox (Saffron Scientific, 1 ppm H_2_O, 1 ppm O_2_).

Room temperature *ex-situ* powder X-ray diffraction (PXD) experiments were conducted with a Bruker D8 powder diffractometer in transmission geometry with spinning sealed capillaries. Diffraction data for phase identification were typically collected over 5° ≤ 2*θ* ≤ 85° with a 0.017° step size and scan times of 1 or 10 h.

Synchrotron PXD data were collected using incident radiation with λ = 0.9533 Å using a Mythen-II detector at the powder diffraction beamline, Australian Synchrotron. Time-resolved *in situ* high temperature measurements were conducted with a flow cell under an atmosphere of 1 bar argon (99.99%) using a Cyberstar hot air blower to heat the quartz capillary from 50 °C to 260 °C at a constant heating rate of 10 °C min^–1^. Data were collected with an exposure time of 150 s at every 10 °C step. Data analysis was performed using the TOPAS 4.2 software package [30].

Hydrogen absorption experiments were performed on an intelligent gravimetric analyzer (IGA, Hiden, Warrington, UK) with samples of ∼50 mg contained in stainless steel sample holders. Hydrogen gas (BOC, 99.98%; Motherwell, UK) at 18 bar was introduced into the reaction chamber and absorption was performed between 20 and 400 °C with a fixed ramp rate of 3 °C min^–1^. The variation in sample mass and temperature with time was recorded.

Thermal behavior of NaH-NaOH samples was analyzed via simultaneous thermogravimetric-differential thermal analysis with coupled mass spectrometry (TG-DTA-MS; Netzsch STA 409 coupled to a Hiden Analytical HPR20 mass spectrometer; Selb, Germany) in an Ar-filled recirculating glovebox (MBraun UniLab; Garching, Germany, 1 ppm H_2_O, 1 ppm O_2_). Samples of *ca*. 20 mg were loaded into alumina pans and heated to 673 K under a flow of Ar gas at a rate of 5 °C min^–1^ in order to achieve complete decomposition of the samples.

## 4. Conclusions

In summary, the Na-O-H reversible hydrogen storage system has been examined in detail. The (de)hydrogenation behavior of the Na-O-H system was investigated by means of IGA measurements, simultaneous TG-DTA-MS, XRD, and *in-situ* synchrotron PXD. Na_2_O starts to absorb hydrogen at temperatures slightly above ambient (30–50 °C) under a relatively low hydrogen pressure (18 bar). The hydrogenated products, NaH and NaOH, release hydrogen at a lower temperature than the binary hydride NaH. Moreover, differential thermal analysis shows an endothermic event near 170 °C in the NaH-NaOH system that cannot be associated with the dehydrogenation reaction of the mixture or with phase transitions of either of the individual components. *In-situ* synchrotron PXD results show that NaOH forms a solid solution with NaH that persists until 240 °C. The NaH and Na(H,OH) cubic phases demonstrate a large volume expansion in the temperature range of 160−240 °C. The resulting predominantly *intraphase* interaction of protic hydrogen and hydridic hydrogen provides a likely driving force for the subsequent dehydrogenation in the hydride-hydroxide system.
